# Plastic or metal stents for benign extrahepatic biliary strictures: a systematic review

**DOI:** 10.1186/1471-230X-9-96

**Published:** 2009-12-17

**Authors:** Petra GA van Boeckel, Frank P Vleggaar, Peter D Siersema

**Affiliations:** 1 Department of Gastroenterology and Hepatology, University Medical Center Utrecht, Utrecht, The Netherlands

## Abstract

**Background:**

Benign biliary strictures may be a consequence of surgical procedures, chronic pancreatitis or iatrogenic injuries to the ampulla. Stents are increasingly being used for this indication, however it is not completely clear which stent type should be preferred.

**Methods:**

A systematic review on stent placement for benign extrahepatic biliary strictures was performed after searching PubMed and EMBASE databases. Data were pooled and evaluated for technical success, clinical success and complications.

**Results:**

In total, 47 studies (1116 patients) on outcome of stent placement were identified. No randomized controlled trials (RCTs), one non-randomized comparative studies and 46 case series were found. Technical success was 98,9% for uncovered self-expandable metal stents (uSEMS), 94,8% for single plastic stents and 94,0% for multiple plastic stents. Overall clinical success rate was highest for placement of multiple plastic stents (94,3%) followed by uSEMS (79,5%) and single plastic stents (59.6%). Complications occurred more frequently with uSEMS (39.5%) compared with single plastic stents (36.0%) and multiple plastic stents (20,3%).

**Conclusion:**

Based on clinical success and risk of complications, placement of multiple plastic stents is currently the best choice. The evolving role of cSEMS placement as a more patient friendly and cost effective treatment for benign biliary strictures needs further elucidation. There is a need for RCTs comparing different stent types for this indication.

## Background

Benign biliary strictures occur most frequently as a consequence of a surgical procedure of the gallbladder, mainly cholecystectomy, or common bile duct (CBD) [1]. Other causes include inflammatory conditions, such as chronic pancreatitis and sclerosing cholangitis [2]. In addition, cholelithiasis, sphincterotomy and infections of the biliary tract may also lead to a stricture [3]. Benign strictures of the biliary tract are associated with a broad spectrum of signs and symptoms, ranging from subclinical disease with mild elevation of liver enzymes to complete obstruction with jaundice, pruritus and cholangitis, and ultimately biliary cirrhosis [4].

A bilio-digestive anastomosis, or a percutaneously or endoscopically performed dilation with or without stent placement are the most commonly used treatment options for benign biliary strictures[5]. Stent placement in the CBD is an increasingly being used alternative to surgery. Several reports on the nonsurgical management of benign biliary strictures with stents have shown results which are equal to those obtained by surgery [6-12]. The endoscopic management typically consists of dilation and insertion of one or more plastic stents followed by elective stent exchange every 3 months to avoid cholangitis caused by stent clogging [4,13]. An increasing number of plastic stents will progressively dilate a stricture in the CBD or the papilla. The major disadvantages of this method are the need for multiple invasive procedures and the morbidity caused by stent dysfunction resulting in recurrent jaundice and cholangitis.

In malignant biliary strictures, uncovered self-expanding metal stents (uSEMS) have been shown to have a longer stent patency than plastic stents, mainly because of their larger diameter [4,14]. Nonetheless, long-term stent patency is a limiting factor with uSEMS as well, as these devices may obstruct due to epithelial hyperplasia and tissue ingrowth through the stent meshes [15-17]. This process of epithelial hyperplasia causes embedding of the stent into the bile duct mucosa, making removal of uSEMS difficult or even impossible [18]. These drawbacks limit the use of uSEMS in the treatment of benign biliary strictures.

Only limited data comparing the efficacy and safety of different biliary stent types for benign biliary strictures are available. We therefore performed a systematic review of the current literature to assess technical and clinical success, and complications of different stent types for this indication.

## Methods

### Systematic search

**Figure 1:**
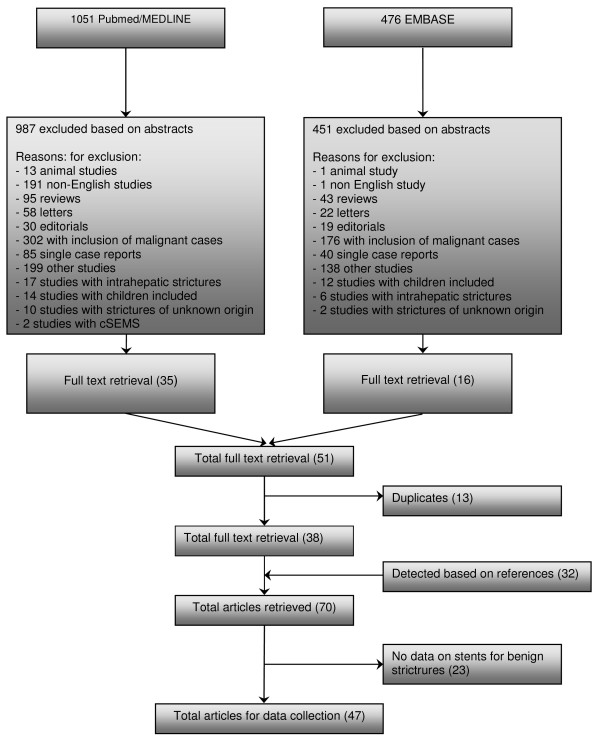
**Flowchart of search history on stents for benign extrahepatic biliary strictures**.

A systematic search of PubMed between January 1966 and March 2008 and EMBASE between January 1980 and March 2008 was performed. In PubMed, the MeSH headings 'cholestasis' and 'obstructive jaundice' were used in combination with the MeSH heading 'stent'. In EMBASE a similar search using the same headings was performed. We detected 1051 abstracts in PubMed and 476 abstracts in EMBASE and these 1527 abstracts were evaluated. All studies reporting on biliary stent placement in patients with benign strictures were included. Non-English language studies, letters, editorials, reviews, animal studies, single case reports, studies with data on covered self-expandable metal stents (cSEMS), studies with results on intrahepatic strictures, studies with strictures of unknown origin and studies in patients with malignant strictures or children were excluded. This resulted in 51 abstracts being retrieved as full text. Thirteen studies were excluded because they were duplicates and 23 studies because they contained no data on stent placement for benign biliary strictures. Another 32 studies were added after manual searching of references in the selected studies. Finally, 47 studies were retrieved for data extraction (Figure 1).

### Data extraction

Data on study design, number of patients, etiology and location of the stricture, route of stent placement, stent type, follow-up time, previous treatment, median stenting time, technical and clinical success rates, patency rate, complications, stricture recurrence and mortality were extracted.

### Definitions

- Stenting time: the time between stent placement and removal. Stenting time in patients treated with uSEMS was defined as the time between stent placement and the moment that further treatment was indicated because of stent obstruction.

- Technical success: technically successful stent placement.

- Clinical success: no need for further treatment after stent placement, relief of symptoms and/or significant decrease in bilirubin level after stent placement.

- Complication: adverse event after stent placement, such as cholangitis, pancreatitis, stent migration or hemorrhage.

- Mortality: procedure-related and stent-related death.

### Statistics

The following data were pooled using a fixed effect model: stenting time, technical success rate, clinical success rate, complications and mortality. The number of patients with a single plastic stent, multiple plastic stents and uSEMS were plotted against clinical and technical success rates, resulting in funnel plots, a statistical method used for assessing publication bias [19]. If publication bias is not present, a funnel plot is expected to be roughly symmetrical. The underlying idea is that studies with the largest number of patients estimate clinical and technical success rates more accurately than studies with fewer patients. As it may be difficult to establish publication bias by visual inspection [20], we used the Mann-Whitney U test and Spearman's rank correlation test to determine a correlation between technical and clinical success rates per stent type and the number of patients. A p-value < 0.05 was considered statistically significant. SPSS software, version 15, (Inc., Chicago, Illinois, USA) was used to perform the statistical analysis.

## Results

### Study types

From the 47 selected studies, data on outcome of biliary stenting in 1116 patients were extracted (Table 1, 2). Of these, 24 studies reported on single plastic stents [2,21-42], 6 on multiple plastic stents [43-48] and17 on uSEMS [15,16,49-63]. A single plastic stent was compared with multiple plastic stents in one non-randomized study [64]. The remaining studies were all case series, of which 33 were retrospective[16,21-29,31,35-37,39,41-44,47,49-51,53-55,59,60,62,64] and 14 prospective in design[15,30,32,34,38,40,45,48,52,56-58,61,63]

**Table 1:** Case series with uncovered SEMS (uSEMS) for benign biliary strictures

Author	Year	N	Age (years (range))	Women	Intervention	Route	Etiology stricture	Location obstruction
**Prospective studies**								
Yamaguchi et al [63]	2006	8	median 65,7 (42-78)	0	Streckerstent (2)	ERCP	chronic pancreatitis	CBD
O Brien et al [58]	1998	8	median 59 (26-88)	unknown	Wallstent	ERCP	postoperative/endoscopic (5)	hilair (3)
							chronic pancreatitis (2)	proximal (5)
							idiopathic (1)	
Deviere et al [15]	1994	20	mean 45 (27-61)	4	Wallstent	ERCP	chronic pancreatitis	CBD
Mygind et al (75)	1993	2	unknown	2	Z stent	PTC	post operative	CBD (1)
								CBD and anastomosis (1)
Maccioni et al [56]	1992	18	mean 60 (22-76)	8	Z stent (17)	PTC	post operative	anastomosis (13)
					Wallstent (1)			CBD (5)
Foerster et al [52]	1991	7	median 60 (49-80)	5	Wallstent	ERCP (6)	postoperative	anastomosis (2)
						PTC (1)		CBD (5)
								hepatoduodenal fistel (1)
**Retrospective studies**								
van Berkel et al [62]	2004	13	mean 56 (40-79)	4	Wallstent	ERCP	chronic pancreatitis	CBD
Roumilhac et al [60]	2003	12	unknown	unknown	Metal stent (12)	ERCP	post OLT	anastomosis (11)
Eickhoff et al [51]	2003	6	median 38 (29-60)	1	Wallstent	ERCP	chronic pancreatitis	CBD
Kahl et al [55]	2002	3	mean 48 (21-81)	1	Wallstent (3)	ERCP	chronic pancreatitis	CBD
Bonnel et al [49]	1997	25	mean 64 (35-86)	13	Z stent	PTC	postoperative	CBD (8)
								anastomosis (17)
Rieber et al [59]	1996	8	mean 42 (17-66)	3	Palmaz stent	PTC	post OLT	anastomosis (5)
								nonanastomotis (3)
Hausegger et al [53]	1996	20	mean 62 (36-83)	7	Wallstent	PTC	chronic pancreatitis (7)	anastomosis (4)
							fibrous papillary stenosis (2)	CBD (16)
							psc (1)	
							post operative (10)	
Chu et al [50]	1994	2	unknown	unknown	Z stent	PTC	post operative	hilair (1)
								CBD (1)
Ivancev et al [54]	1992	2	66 and 41	2	Z stent	PTC	post operative	anastomosis (1)
								CBD (1)
Rossi et al [16]	1990	17	mean 60 (22-76)	7	Z stent	PTC	postoperative	anastomosis (13)
								CBD (4)

**Table 2:** Case series with multiple plastic stents and single plastic stents for benign biliary strictures

Author	Year	N	Age (years (range))	Women	Intervention	Route	Etiology stricture	Location obstruction
**Prospective studies**								
Holt et al [32]	2007	53	48,5 (37-61)	32	single plastic stent	ERCP	post OLT	anastomosis
Graziadei et al [30]	2006	84	53,5	21	single plastic stent	ERCP	post OLT	anastomosis (65)
								non anastomosis (19)
Pozsar et al [48]	2005	20	mean 61,3 (36-81)	18	multiple plastic stents	ERCP	post sphincterectomy	distal CBD
Kuzela et al [45]	2005	43	mean 50,3 (37-82)	25	multiple plastic stents	ERCP	post operative	hilair
Kahl et al [34]	2003	61	median 47 (21-81)	15	single plastic stent	ERCP	chronic pancreatitis (61)	CBD
Tocchi et al [38]	2000	20	mean 57	10	single plastic stent	ERCP	post operative	CBD (3)
								hilair (17)
van Milligen et al [40]	1997	16	median 43 (17-69)	8	single plastic stent	ERCP	psc	CBD (10)
								hiliar (6)

**Retrospective studies**								

Pasha et al [47]	2007	25	mean 46,7 (28-59)	4	multiple plastic stents	ERCP	post OLT	anastomosis
Elmi et al [28]	2007	15	52 year (42-68)	9	single plastic stent	ERCP	post OLT	anastomosis
Akay et al [21]	2006	11	42 (17-60)	6	single plastic stent	ERCP	post OLT	anastomosis
Sharma et al [37]	2006	8	median 42 (20-61)	3	single plastic stent	ERCP	idiopathic	CBD (6)
								hilair (2)
Alazmi et al [22]	2006	143	unknown	unknown	single plastic stent	ERCP	post OLT	anastomosis
Zoepf et al [42]	2005	7	median 55 (45-65)	unknown	single plastic stent	ERCP	post OLT	anastomosis
Cahen et al [25]	2005	58	median 54 (19-85)	10	single plastic stent	ERCP	chronic pancreatitis	CBD
Catalano et al [64]	2004	46	mean 48 (30-71)	11	1 plastic stent (34)	ERCP	chronic pancreatitis	CBD
					multiple plastic stents (12)			
Morelli et al [46]	2003	25	mean 48 (18-72)	9	multiple plastic stents		post OLT	anastomosis
Hisatsune et al [31]	2003	19	45 (14-67)	9	single plastic stent	ERCP	post OLT	anastomosis
Eickhoff et al [27]	2001	39	mean 54,7 (32-81)	7	single plastic stent	ERCP	chronic pancreatitis (39)	CBD
Bourke et al [43]	2000	6	mean 53 (20-64)	3	multiple plastic stents	ERCP	post sphyncterectomy	ampullary
Khandekar et al [44]	2000	17	median 50 (17-68)	13	multiple plastic stents	ERCP	post sphyncterectomy (10)	CBD (14)
							papillotomy (2)	other (3)
							post operative (3)	
Vitale et al [41]	2000	25	mean 46,7 (36-89)	7	single plastic stent	ERCP	chronic pancreatitis	CBD
Kiehne et al [35]	2000	14	(36-89)	2	single plastic stent		chronic pancreatitis	CBD
Farnbacher et al [29]	2000	31	50 (24-71)	3	single plastic stent	ERCP	chronic pancreatitis	CBD
Rossi et al [36]	1998	15	mean 44 (28-55)	6	single plastic stent	ERCP	post OLT (15)	anastomosis
De Masi et al [26]	1998	53	unknown	unknown	single plastic stent	ERCP	iatrogenic (39)	CBD (20)
							gallstones (8)	hilair (30)
Aru et al [23]	1997	8	mean 44	7	single plastic stent	ERCP	post operative	CBD (7)
								hilair (1)
van Milligen et al [39]	1996	25	median 42 (21-74)	13	single plastic stent	ERCP	psc	CBD (19)
								hilair (3)
Itani et al [33]	1995	5	unknown	unknown	single plastic stent	ERCP	chronic pancreatitis	CBD
Barthet et al [24]	1994	19	mean 49	1	single plastic stent	ERCP	chronic pancreatitis	CBD
Deviere et al [2]	1990	25	mean 42 (34-69)	1	single plastic stent	ERCP	chronic pancreatitis	CBD

### Patients

Fourty seven studies evaluated 786 patients treated with a single plastic stent (7-11.5 Fr.), 148 with multiple plastic stents (10-11.5 Fr.) and 182 with uSEMS.

Indications for stent placement included a biliary stricture secondary to liver transplantation (n = 417, 37%), chronic pancreatitis (n = 380, 34%), surgery (n = 170, 16%), and other causes (n = 149,13%).

Most strictures were located in the CBD (47%), followed by anastomotic strictures (40%), hilar strictures (11%) and other locations (2%) (Table 3, 4).

**Table 3:** Results on route, previous treatment, treatment time, technical success, clinical success and complications in case series with uncovered SEMS (uSEMS) for benign biliary strictures

Author	Intervention	Follow up (range)	Previous treatment	Technical success	Clinical succes	Treatment time Stentpatency	Total complications
**Prospective studies**							

Yamaguchi et al [63]	Streckerstent (2)	> 5 years (7.4 year)	plastic stent placement	100%	62,50%	unknown	25%
	Wallstent (6)						
O Brien et al [58]	Wallstent	mean 64,5 months (26-81)	plastic stent placement (5)	100%	unknown	median 35 months (7-57)	75
Tesdal et al [69]	Wallstent (11)	mean 63,8 months	balloon dilatation (19)	100%	unknown	mean 30,2 months	64,50%
	Palmazstent (9)	median 80,5 (2-116)					
	Streckerstent(4)						
Deviere et al [15]	Wallstent	mean 33 months (24-42)	plastic stent placement (11)	100%	90%	3 and 6 months (2/20)	10
Mygind et al (75)	Z stent	4 and 7 months	balloon dilatation	100%	100%	unknown	unknown
Maccioni et al [56]	Z stent (17)	mean 37 months (30-41)	percutaneous dilatation	83,30%	55,50%	unknown	38,80%
	Wallstent (1)						
Foerster et al [52]	Wallstent	mean 32,7 weeks (21-53)	laparotomy (2)	100%	100%	8 months until now	14%

**Retrospective studies**							

van Berkel et al [62]	Wall stent	mean 50 months (6 d -86 months)	none	100%	69%	60 months	15,40%
Roumhilac et al [60]	SEMS (12)	median 37 months (18-53)	plastic stent treatment for 1 year	100%	100%	no stent obstruction	unknown
Eickhoff et al [51]	Wallstent	median 58 months (22-29)	plastic stent placement	100%	unknown	median 20 months (10-38)	83,40%
Kahl et al [55]	Wallstent (3)	median 37 months (18-53)	plastic stent treatment for 1 year	100%	100%	no stent obstruction	unknown
Bonnel et al [49]	Z stent	mean 55 months (9-84)	surgery (17)	18 one approach	72%		36%
			T tube (8)	7 two approaches			
Rieber et al [59]	Palmaz stent	mean 18 months (1,5-43)	balloon dilatation	100%		62% occlusion	
			post PTBD			occlussion time 1,5-2,5-24 months	
Hausegger et al [53]	Walsltent	mean 31,2 months (3-78)	balloon dilatation	100%	unknown	73% (6 months)	50,00%
						38% (36 months)	
						19% (end follow up)	
						3-3-3-4-5-11-24-2-36-55	
Chu et al [50]	Z stent	unknown	plastic stents	unknown		0%	
			PTBD				
Ivancev et al [54]	Z stent (2)	9 and 14 months	balloon dilatation	100%	50%	50% (5 months)	50%
Rossi et al [16]	Z stent	mean 8 months (4-12)	baloon dilatition	100%	82,40%	unknown	11,80%

**Table 4:** Results on route, previous treatment, treatment time, technical success, clinical success and complications in case series with multiple plastic stents and single plastic stents for benign biliary strictures

Author	Intervention	Follow up (range)	Previous treatment	Technical success	Clinical succes	Treatment time Stentpatency	Total complications
**Prospective studies**							
Holt et al [32]	single plastic stent	18 months	balloon dilatation	92%	69%	11,3 months (7-14)	69,70%
Graziadei et al [30]	single plastic stent	mean 39,8 (0,3-98)	balloon dilatation	unknown	77% anastomosis	unknown	5-424 procedures
					0% non anastomosis		
Pozsar et al [48]	multiple stent placement	mean 61,3 (36-81)	dilatation	unknown	90%	median 9 months (3-22)	37,70%
Kuzela et al [45]	multiple stent placement	median 16 months (1-42)	none	100%	100%	1 year	12%
		after stent placement				(planned)	
Tocchi et al [38]	single plastic stent	mean 89,7 months	none	100%	80%	unknown	0%
Kahl et al [34]	single plastic stent	median 40 months (18-66)	none	100%	31,1% (1 year)	1 year (19)	34,40%
					26,2% (40 months)	rest unknown	
van Milligen et al [40]	single plastic stent	median 19 months (7-27)	none	100%	81%	median 9 days	7%

**Retrospective studies**							
Pasha et al [47]	multiple plastic stent	median 21,5 months (5,4-31,2)	diliatation	unknown	88% (intend to treat)	median 4,6 months (1,1-11,9)	27%
Elmi et al [28]	single plastic stent	535 days (22-1301)	balloondilatation	Unknown	87%	192 days (18-944)	22,2% (procedure)
			sphincterectomy				
Akay et al [21]	single plastic stent	22 months (SD 13 months)	balloondilatation	75%	55%	3 months (6)	12%
						6 months (1)	
						9 months (1)	
						12 months (3)	
Sharma et al [37]	single plastic stent	median 19 months (4-52)	balloondilatation	100%	100%	median 19 months	18%
Alazmi et al [22]	single plastic stent	mean 28 months (1-114)	balloondilatation	6,60%	82%	unknown	unknown
Zoepf et al [42]	single plastic stent	median 9,5 months (1-36)	sometimes dilatation	100%	85,60%	median 8 months (2-26)	18,60%
Cahen et al [25]	single plastic stent	median 45 months (0-182)	sphincterectomy	100%	38%	median 274 days (3-2706)	52%
			pancreatic duct stenting				
Catalano et al [64]	single plastic stent (34)	mean 4,2 years (1 plastic stent)	unknown	100%	24% 1 stent	21 months	42,7% (single plastic stent)
	multiple plastic stent (12)	mean 3,9 years (mulitple stents)			92% multiple stents	14 months	8,3% (multiple plastic stent)
Morelli et al [46]	multiple plastic stent	mean 54 weeks (5 wks - 103 mo)	diliatation	88%	90%	unknown	3,70%
Hisatsune et al [34]	single plastic stent	mean 26 months(15-44)	none	79%	93%	mean 637 days (487-933)	43%
Eickhoff et al [31]	single plastic stent	median 58 months (2-146)	balloon dilatation	100%	31%	mean 9 months (1-144)	43%
			nasobiliary drainage				
Bourke et al [43]	multiple plastic stent	median 26,5 months (24-32)	dilatation	unknown	100%	median 12,5 months	33%

**Author**	**Intervention**	**Follow up (range)**	**Previous treatment**	**Technical success**	**Clinical succes**	**Treatment time** **Stentpatency**	**Total complications**
Khandekar et al [44]	multiple plastic stent	median 720 days	sometimes dilatation	Unknown	100%	median 140 days (30-1080)	unknown
Vitale et al [41]	single plastic stent	32 months (13-76)	balloon dilatation	Unknown	80%	mean 13,3 months	unknown
Khiene et al [35]	single plastic stent	1-5 years	none	100%	7,40%	unknown	85,70%
Farnbacher et al [29]	single plastic stent	24 months (2-76)	none	100%	13%	24 months (2-76 months)	72%
Rossi et al [36]	single plastic stent	1 year	dilatation	100%	83,30%	1 year	33,30%
De Masi et al [26]	single plastic stent	6-84 months	unknown	Unknown	71,40%	24 months	52,70%
Aru et al [23]	single plastic stent	unknown	unknown	100%	25%	unknown	unknown
van Millegen et al [39]	single plastic stent	mean 29 months (2-120)	dilatation nasobiliary drain	84%	76%	1 stent period (17)	30,5%(procedure)
						2 stent period (2)	
						3 stent period (3)	
Itani et al [33]	single plastic stent	mean 7 months	dilatation	100%	80%	4 months (2)	unknown
						1 change 4 months (2)	
						15 months (1)	
Barthet et al [24]	single plastic stent	mean 18 months (13-48)	none	100%	42%	mean 10 months	10,50%
Deviere et al [2]	single plastic stent	mean 14 months (4-72)	dilatation	100%	12%	unknown	72%

In the majority of patients with chronic pancreatitis, a single plastic stent was placed (85%), followed by uSEMS (15%) and multiple plastic stents (0%). Similarly, single plastic stents were placed in 82% of patients with a biliary stricture after liver transplantation, followed by uSEMS (22%) and multiple plastic stents (13%). In patients with a biliary stricture after a surgical procedure uSEMS (50%) were placed most frequently followed by multiple plastic stents (35%) and a single plastic stent (15%).

### Comparison between different stent types

The median stenting time was not different between multiple plastic stents (11.3 (range 4.6-13) months) and single plastic stents (10.5 (0.3-24) months). Median stenting time was 20 (4.5-60) months for uSEMS.

**Figure 2:**
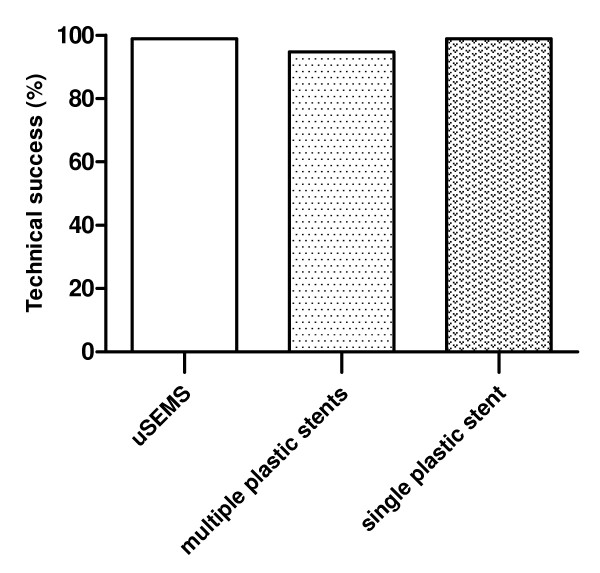
**Technical success of uncovered SEMS (uSEMS), multiple plastic stents and single plastic stents for benign biliary strictures**.

The technical success rate was not different between different stent types (98,9% for uSEMS and 94.8% for single plastic stents, 94.0% for multiple plastic stents) (Figure 2).

**Figure 3:**
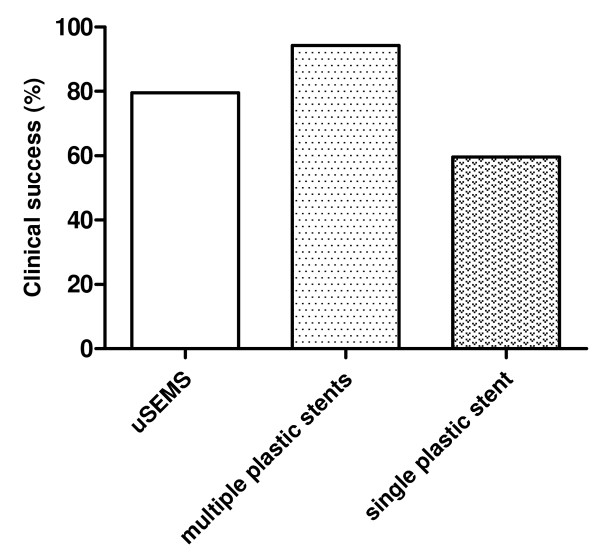
**Clinical success of uncovered SEMS (uSEMS), multiple plastic stents and single plastic stents for benign biliary strictures**.

The clinical success rate for all patients was highest after placement of multiple plastic stents (94,3%) followed by uSEMS (79.5%) and single plastic stents (59,6%) (Figure 3). Clinical success rate in chronic pancreatitis patients was highest for uSEMS (80.4%) and lowest for single plastic stents (35.9%). Multiple plastic stents had the best clinical performance for strictures following liver transplantation (89.0%) and surgery (81.3%), whereas uSEMS (69% and 62.3%, respectively) showed the worst clinical results in these situations (Table 5).

**Table 5:** Overview of technical and clinical success of uncovered SEMS (uSEMS), multiple plastic stents and single plastic stents for benign biliary strictures

	Single plastic stent		USEMS		Multiple plastic stents	
	**Technical success**	**Clinical success**	**Technical success**	**Clinical success**	**Technical success**	**Clinical success**
	**(mean)**	**(mean)**	**(mean)**	**(mean)**	**(mean)**	**(mean)**

All indications	94,10%	61.3%	98,50%	62,40%	97,60%	87,50%
Post operative	86,6,%	64,90%	97,60%	59,60%	100	87,60%
Chronic pancreatitis	100%	36,60%	100%	80,40%	NA	NA
Post OLT	97,20%	81%	100%	50%	88%	89%

**Figure 4:**
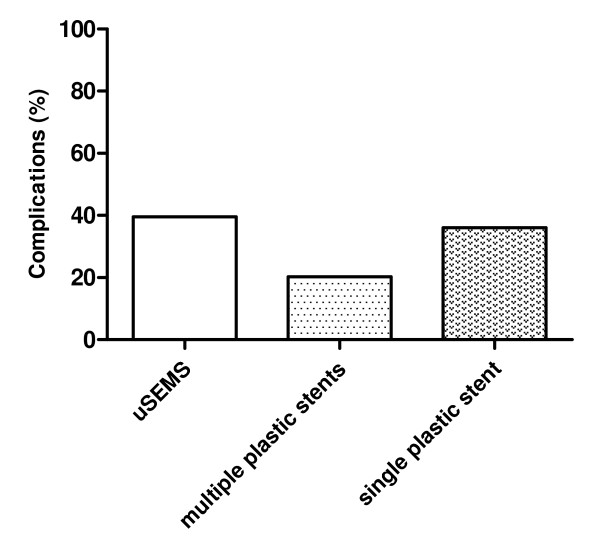
**Complications of uncovered SEMS (uSEMS), multiple plastic stents and single plastic stents for benign biliary strictures**.

Complications occurred most frequently with uSEMS (39.5%), followed by a single plastic stent (36.0%) and multiple plastic stents (20.3%) (Figure 4). The most frequently reported complications included cholangitis, pancreatitis, stent migration and hemorrhage.

No stent-related mortality was reported with placement of multiple plastic stents, whereas 7 (0.9%) patients died as a consequence of single plastic stent placement. Following uSEMS placement, 2 (1.1%) patients died of a stent-related cause. In all these cases, the cause of death was a septic complication due to cholangitis.

### Publication bias

**Figure 5:**
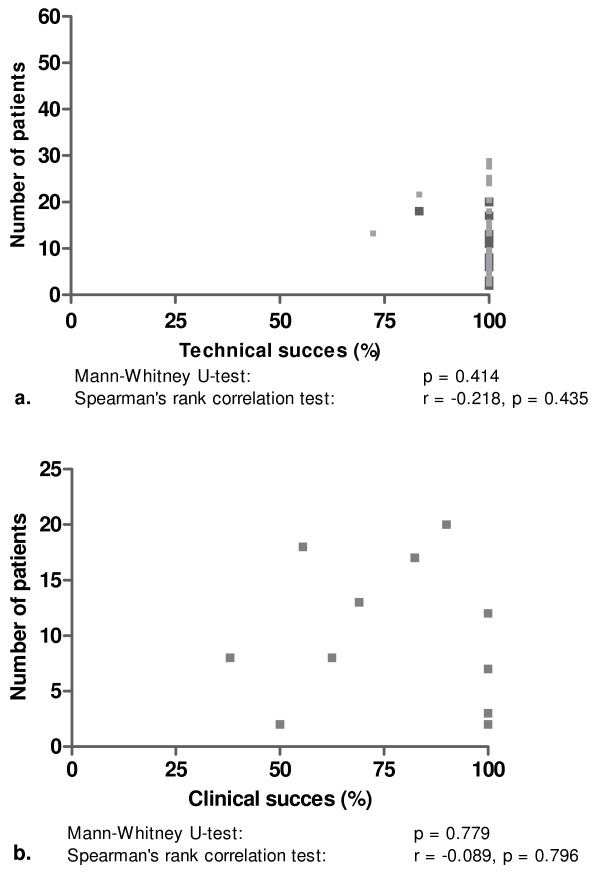
**Numbers of patients with a benign biliary stricture vs. reported results for technical success (a) and clinical success (b) of uncovered self-expanding metal stent placement**.

Plotting the total number of patients with uSEMS against technical and clinical success showed that publication bias was not present (Figure 5). This was confirmed with Spearman's rank correlation test for technical (r-0.218, p = 0.435) and clinical success (r-0.089, p = 0.796) against the number of included patients. The same was found when technical success and clinical success rates in publications with ≤ 8 or >8 patients were compared (p = 0.414 and p = 0.779, respectively).

**Figure 6:**
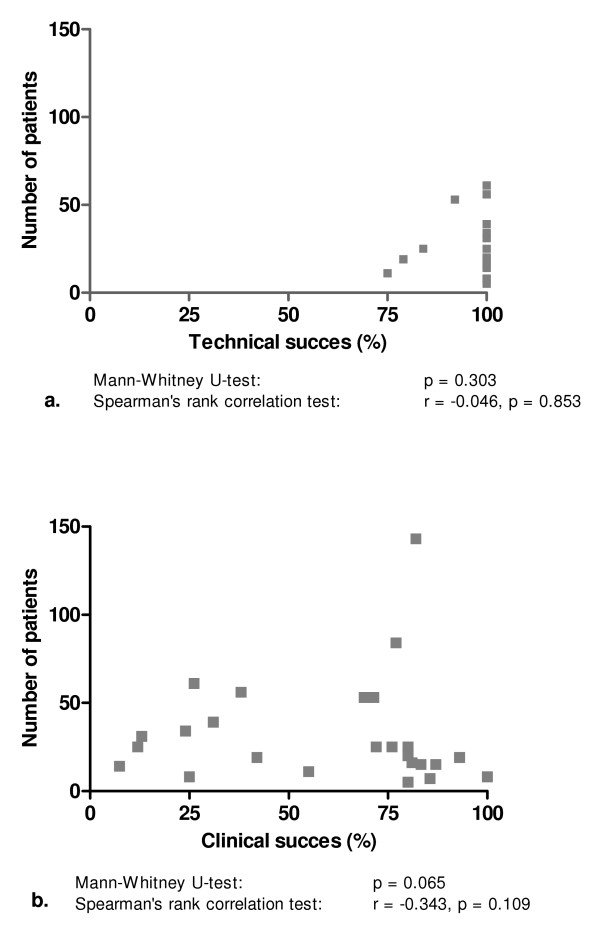
**Numbers of patients with a benign biliary stricture vs. reported results for technical success (a) and clinical success (b) of single plastic stent placement**.

We also plotted the number of patients with a single plastic stent against technical and clinical success and again found no evidence of publication bias (Figure 6). Similarly, no evidence of bias was found when the clinical success in publications with ≤ 20 or >20 included patients were compared (p = 0.065). For clinical success, this was confirmed with Spearman's rank correlation test (r-0.343, p = 0.109). For technical success, however, Spearman's rank correlation test suggested publication bias (r-0.046, p = 0.109). On the other hand, no evidence of bias was found when publications with ≤ 20 or >20 patients were compared (p = 0.303).

As the number of publications on multiple plastic stents (n = 6) in benign biliary stricture was low, it was not possible to make funnel plots for this stent type.

## Discussion

This review shows that the most optimal nonsurgical treatment of benign extrahepatic biliary strictures has been demonstrated with multiple plastic stent placement. These results confirm that dilation with a large diameter dilator, i.e. multiple plastic stents, for a prolonged period is the most effective way to relieve benign strictures. It is however important to note that these results were mainly based on case series with often small patient numbers included.

Complication rates were also lowest for multiple plastic stents, followed by single plastic stents and uSEMS. The low complication rate of multiple plastic stents is most likely due to the practice of exchanging multiple plastic stents at 3-months intervals. This was found to be uncommon after single plastic stent placement. In the latter, cholangitis as a result of stent clogging occurred more frequently. Due to their larger luminal diameter, placement of uSEMS seems an attractive alternative for single or multiple plastic stents in benign biliary strictures, however uSEMS have the disadvantage that tissue hyperplasia through uncovered stent meshes may occur, leading to stent obstruction [15,16,65]. Based on clinical success and complication rates, placement of multiple plastic stents has therefore still the best treatment profile for treatment of benign biliary strictures.

Our findings are in line with results of stent placement for specific causes of benign biliary obstruction, particularly those following liver transplantation or a surgical procedure. Only for patients with strictures due to chronic pancreatitis, uSEMS were found to give good results with regard to clinical success. The number of studies that included patients with this indication and were treated with multiple plastic stents was low. The reason for this is likely that biliary obstruction due to chronic pancreatitis often has a protracted course, requiring multiple procedures if plastic stents are used [66].

An exception to the overall poor results of endoscopic treatment with single plastic stents in patients with chronic pancreatitis was reported by Vitale et al. [41], who achieved stricture resolution with single plastic stents in 80% of patients. Calcifications in the pancreatic head were found in only 4 of 25 patients in this study, which may well explain the high success rate. Calcifications in the pancreatic head have been suggested to be a strong predictor of failure of CBD stenting [34]. As these calcifications are often associated with a firm fibrotic component due to the inflammatory reaction in chronic pancreatitis [67], it can be expected that these strictures are more difficult to dilate. Patients with chronic pancreatitis but without calcifications are more likely to have a stricture secondary to edema and to have less pronounced fibrosis. These strictures may subside over time and therefore only require temporary treatment. This explains why single plastic stent placement for CBD strictures in this patient category was found to be successful (78).

It should be noted that the disappointing results of uSEMS placement, particularly in patients with biliary strictures following liver transplantation or a surgical procedure, are probably affected by selection bias. In most studies, the included population consisted of patients in whom the initial treatment, mostly plastic stent placement, had already failed. As a consequence, these patients were probably more difficult to treat and less responsive to dilation.

We found that the median stenting time was not different between multiple and single plastic stent placement (11.3 vs.10.5 months, respectively).uSEMS functioned clinically well for a median time of 20 months (0.5-60) before a reintervention, mostly for stent obstruction, was needed. Reported reinterventions included placement of a new stent within the occluded uSEMS, percutaneous biliary drainage, endoscopic removal of sludge, or surgical or endoscopic removal of the stent.

A problem with uSEMS is that they tend to embed into the mucosa of the CBD, leading to mucosal hyperplasia. This is an unwanted side effect, as removal of uSEMS in this situation is difficult, if not impossible. Removal may however be indicated when uSEMS are malpositioned or obstructed, or have (partially) migrated [18,68]. Recently, cSEMS have been introduced. These devices have the benefit that removal is possible as the risk of embedding into the biliary wall is reduced or even negligible. This capacity combined with the larger diameter of cSEMS makes stepwise dilation, as is performed with multiple plastic stents, unnecessary and may thus reduce the number of procedures [69]. The clinical experience with cSEMS for benign biliary strictures is until now only limited [66,69,69]. cSEMS can achieve a luminal diameter that is comparable to that of multiple plastic stents and uSEMS, but due to their covering have the advantage that fewer procedures for recurrent obstruction are required. In the future, cSEMS are likely to be a more patient-friendly and cost-effective treatment option for benign biliary strictures. Until now, cSEMS placement for benign biliary strictures is still associated with relatively high complication rates (39.6%) [66,69,69]. In our opinion, new covered stents and refinements of existing covered stents are needed before large scale introduction of cSEMS for this indication can be recommended.

This review has several limitations which should be taken into account before concluding that a particular stent type is favorable in patients with a benign biliary stricture. First, no randomized trials and only one comparative trial have been conducted. This may be due to the fact that (multiple) plastic stents have an acceptable technical and clinical success rate in daily clinical practice. Moreover, uSEMS placement has not been shown to be more successful than multiple plastic stents in case series.

Secondly, several types of plastic stents were used in different studies. Results on individual plastic stent types in patients with benign biliary strictures are not available. From trials in patients with malignant biliary strictures, it is however known that different plastic stents types have varying luminal patencies, due to the stent material and/or the stent diameter [70-73]. Particularly, plastic stents with a diameter of 10 French (Fr.) have been shown to be remain patent for a significantly longer period than 8 Fr. stents (median 32 vs. 12 weeks) [71].

Finally, there was a wide variety in treatment protocols in the various studies with plastic stents. In some studies, stent exchange was performed at 3-month intervals, while in other studies stents were only exchanged when they became occluded. Besides, the number of plastic stents used for multiple stenting varied between 2 and 4 among patients. This could both have affected clinical success rates, but also complication rates in patients treated with plastic stents.

The strength of this review is that all available data on the use of plastic stents and SEMS for the treatment of biliary strictures was evaluated. To the best of our knowledge, this is the largest review on the use of different types of stents in patients with a benign biliary stricture, with pooled data on 1116 treated patients. We also showed that the reported results, particularly those of single plastic stents and uSEMS, were not affected by publication bias, making an overestimation of the clinical success rate and/or an underestimation of the complication rate of a particular stent type unlikely.

## Conclusion

In conclusion, this systematic review shows that, based on clinical success and risk of complications, placement of multiple plastic stents is currently the best choice. The evolving role of cSEMS placement as a more patient friendly and cost effective treatment for benign biliary strictures needs further elucidation. There is a need for RCTs comparing different stent types for this indication.

## Competing interests

The authors declare that they have no competing interests.

## Authors' contributions

PB: literature search, data interpretation, writing of the manuscript. FV: data interpretation, manuscript editing. PS: data interpretation, manuscript editing. All authors read and approved the final manuscript.

## Pre-publication history

The pre-publication history for this paper can be accessed here:

http://www.biomedcentral.com/1471-230X/9/96/prepub
